# Long-term effects of a telemedically-assisted lifestyle intervention on glycemic control in patients with type 2 diabetes – A two-armed randomised controlled trial in Germany

**DOI:** 10.1007/s40200-023-01290-6

**Published:** 2023-10-25

**Authors:** Annalena Dunkel, Katja von Storch, Martin Hochheim, Susanne Zank, M. Cristina Polidori, Christiane Woopen

**Affiliations:** 1 NRW Graduate School GROW - Gerontological Research on Well-Being, Faculty of Medicine, Faculty of Human Sciences, University of Cologne, Albertus-Magnus-Platz, 50923 Cologne, DE Germany; 2 Generali Health Solutions GmbH, Cologne, DE Germany; 3 Rehabilitative Gerontology, Faculty of Human Sciences, University of Cologne, Cologne, DE Germany; 4 Ageing Clinical Research, Department II of Internal Medicine, Center for Molecular Medicine Cologne, Faculty of Medicine, University of Cologne, University Hospital Cologne, Cologne, DE Germany; 5 Cologne Excellence Cluster on Cellular Stress- Responses in Aging- Associated Diseases (CECAD), Faculty of Medicine, University of Cologne, University Hospital Cologne, Cologne, DE Germany; 6 Center for Life Ethics, University of Bonn, Bonn, Germany

**Keywords:** Lifestyle modification, Type 2 diabetes, Telemedicine, Self-management, HbA1c

## Abstract

**Purpose:**

Diabetes is considered one of the fastest growing diseases worldwide. Especially in the treatment of type 2 diabetes, lifestyle interventions have proven to be effective. However, long-term studies in real-world contexts are rare, which is why further research is needed. The aim of the present study is to investigate whether effects achieved in the context of a long-term lifestyle intervention can be sustained by patients in the long term.

**Methods:**

In a two-arm randomized trial we compared diabetes care as usual to a lifestyle intervention combining telemedically support and individual needs-based telephone coaching. The study included 151 patients with type 2 diabetes randomized to either the intervention or control group. Intervention Group (IG; N = 86, 80.2% male, mean age: 59.7) received telemedical devices and telephone coaching over a period of 12 months, Control Group (CG; N = 65, 83.1% male, mean age: 58,8) received care as usual. The primary outcome was chance in Hb_A1c_. A follow-up survey was conducted after 24 months.

**Results:**

The intervention group showed significantly better HbA1c- values compared to the control group at both 12 and 24 months (12 M: − 0.52 (-0.73; − 0.32), p < .000; 24 M: − 0.38 (-0.61; − 0.15), p = .001). The strongest change was seen in the first three months, with the best value obtained at 6 months and stable thereafter.

**Conclusion:**

Combined telephone coaching with telemedicine support could lead to better long-term glycemic control in people with type 2 diabetes. In the future, more long-term studies should be conducted in real-world settings and lifestyle interventions should be offered more widely.

## Introduction

Diabetes mellitus (DM) is one of the fastest-growing health problems of the 21st century [1–4]. The International Diabetes Federation (IDF) estimates that DM currently affects approximately 537 million people worldwide between the ages of 20 and 79, representing about 10.5% f the world’s population in this age group. It is expected that up to 783 million people will be affected by 2045 [4].

For Germany, the IDF reports 9.5 million affected people in 2019, which is well above the national estimates and a 25% increase compared to 2017. New prognoses for Germany expect an increase of another 3.6 million people by 2040 [5]. Research on diabetes therapy is hence becoming increasingly relevant in Germany, as can be seen, for example, from the establishment of a national diabetes surveillance system in 2015, funded by the Federal Ministry of Health [6, 7, 8].

In particular, type 2 diabetes (T2DM), which accounts for 90–95% of all diabetes cases in Germany [6], is a disease with high relevance for public health – first due to its prevalence [1, 9–11], second because the major risk factors are potentially preventable [12–15], and last because diabetes is often associated with medical complications and comorbidities. In addition to the direct health burden on those who are suffering from the disease, T2DM also places a major financial burden on the health care system, both direct (e.g., treatment costs, secondary diseases) and indirect (e.g., loss of working hours, etc.) [16–19].

Early detection and treatment are important to prevent sequelae and to treat the disease effectively [20]. Lifestyle interventions, which may include improved self-management, nutritional training, improved physical activity, weight reduction, smoking cessation, and psychosocial counselling, are key elements of treatment [21–28]. This is also reflected in various national and international guidelines that consider lifestyle interventions as basic therapy [25–27]. Despite the clear evidence that this is a beneficial approach, only about 10% of DM patients in Germany receive these types of treatments in addition to medication [29].

Often, lifestyle interventions are limited by various resources such as time, physical distance, or lack of staff capacity that prevent widespread use. Mobile technologies can help create more flexible and cost-effective options in this regard [19, 30–32]. Several reviews and meta-analyses have shown that digitally assisted interventions achieve comparable outcomes to traditional lifestyle interventions [33–35].

While the overall benefits of lifestyle interventions for DM are well documented, there are still too few studies that include long-term effects (> 6 months) and subsequent treatment adherence [30, 33, 36–38]. The present study evaluates a telemedically supported lifestyle intervention *initiative.diabetes*, which is offered by a private health insurance company in Germany. The duration of the initiative.diabetes programme is 12 months, with an additional follow-up survey after 24 months to assess the continuation of the effects in everyday life. Outcomes were assessed at 3, 6, 12, and 24 months. An initial interim evaluation after 3 months already showed some positive trends [39]. The objectives of the present study were to investigate the long-term effects of the initiative.diabetes programme and the long-term maintenance of these effects after 12 and 24 months.

## Participants and methods

### Study design

This study is a two-arm, randomised, prospective study in cooperation with a private German health insurance company (formerly: Central Krankenversicherung AG; since 2020: Generali Deutschland Krankenversicherung AG), which offers a telemedically supported lifestyle programme called *initiative.diabetes* (described in the next section). As part of the study, the intervention group participated in this programme for 12 months, while the control group received only the usual care. In the one-year follow-up phase, both groups received only the usual care. Usual Care means standard treatment by the general practitioner oriented on the Clinical Practice Guidelines [15] of the German Diabetes Society (DDG) together with the German Society for Internal Medicine (DGIM), which is based on the National Treatment Guideline (NVL) “Type 2 Diabetes” [40]. The detailed treatment was not assessed and may include the entire treatment spectrum depending on treatment preferences of the patient and the physician. Using questionnaires, we only excluded participation in another health care program.

The analysis of the data was blinded. The study took place from March 2017 to July 2020 and has been approved by the Ethics Committee of the Medical Faculty of the University of Cologne (Project ID: 17–021) and registered with the German Clinical Trials Registry (DRKS00013737).

### Recruitment of participants

Recruitment took place throughout Germany from March to May 2017. All of the 315,000 insured members of Central Krankenversicherung AG were screened for inclusion criteria and randomised, into either an intervention group (IG) or a control group (CG) in a ratio of approximately 1.5:1. The unequal weighting was chosen because the randomisation took place before making contact, based on the assumption that the rate of acceptances in the control group would be higher and thus both groups would be as equal in size as possible. The inclusion criteria were met by individuals aged between 40 and 67 who were diagnosed with diabetes mellitus type 2 according to the official diagnosis criteria of T2DM (ICD-10 code E11). People who did not speak the German language, pregnant women, people undergoing cancer treatment or suffering from other life-threatening diseases, people with cognitive or mobility impairments, and people in need of long-term care were excluded. The patients’ medical histories were verified via the insurance data. A total of 2,441 people met the inclusion criteria.

After randomisation, all potential participants were contacted by mail in three consecutive waves at intervals of three to five months (May 2017 to January 2018) to request enrollment in the study. 298 persons initially agreed to participate. All participants were informed about the collection and processing of their data for study purposes and consented to it. Afterwards, they received the baseline questionnaire. Individuals assigned to the intervention group also received a personal online code to register for the programme *initiative.diabetes*. In total, 191 people answered the baseline questionnaire and 151 of them could be included in the intention-to-treat population of the study.

### Programme initiative.diabetes

The programme *initiative.diabetes* is a structured lifestyle intervention that combines telemonitoring with individual and needs-based telephone coaching by health specialists or diabetes coaches. The programme runs over a period of 12 months.

Initially, all participants in the programme received a tablet PC, a pedometer, and a blood glucose meter for telemonitoring. All devices are connected via Bluetooth, which automatically transfers the data from the glucometer and pedometer to the tablet computer. This data is also available to the diabetes coach, who supervises the patient over a period of one year. The devices serve both as a motivation and as a continuous feedback instrument for the patients. By visualising real-time data, the devices help participants understand the direct impact of their health behaviour on their blood glucose levels [41].

The individual and needs-based telephone coaching is based on Prochaska’s transtheoretical model and is divided into different stages [42–44]. The focus of the initial consultation is on establishing an empathic relationship between the patient and the coach. In addition, key problems are identified and initial agreements on individual approaches are made. An individual HbA1C target is agreed upon between each patient and coach. This is followed by a six-month intensive coaching phase with at least one phone call per month and a six-month stabilisation phase with coaching sessions every 6 to 12 weeks. Depending on individual needs, the frequency can be increased in both phases. Coaching includes several modules that address key T2DM issues, such as nutrition, physical activity, self-monitoring, medication, emergency management, clinical management, and stress management. The focus in all modules is on the development of individual routines adapted to personal daily life and their long-term establishment. The data transmitted daily from the pedometer and the blood glucose meter serve as a basis for feedback and to increase self-control and self-management. During the study, patients continued to be treated by their physicians, as *initiative.diabetes* is not a substitute for the usual medical care, but supports it.

### Data collection and measurements

The data collection phase of the study ran from May 2017 to July 2020 in several waves and contains a total of five measurement time points: The baseline measurement (T0), two interim measurements at 3 months (T1) and 6 months (T2), the final measurement at the end of the intervention at 12 months (T3), and a follow-up measurement at 24 months (T4).

Data collection was conducted through a variety of channels. On the one hand, various data and questionnaires were collected via online questionnaires, which were done through patient self-report. In addition, data were collected via the health care provider of the telephone coaching and via the company controlling of the health insurance company.

Baseline characteristics including demographic data, medication, health history, comorbidities as well as medical parameters were determined by the use of a questionnaire as well as through the patient data provided by the insurance company. The definition of comorbidities follows the CoDim study [17, 45].

The Hb_A1c_ levels were recorded by the patients’ physicians and served as primary outcomes in this study. The Hb_A1c_ value describes the mean blood glucose concentration of the last two to three months and is considered to be one of the gold standards [46–48].

Secondary outcomes are body mass index (BMI), physician contacts, costs for antidiabetics, physical activity and use of technology. Body mass index was calculated using height and weight (weight in kg/(height in m)²). Height and weight were recorded by the telephone coach for the intervention group and by questionnaire for the control group. During the follow-up survey, height and weight were recorded by questionnaire for both groups.

The number of physician contacts and the costs for antidiabetic drugs were determined by the controlling department of the health insurance company. In contrast to the parameters Hb_A1c_ and BMI, which were each recorded at specific points in time and represent a snapshot, periods (P) of one year each were recorded for these parameters. These represent the year before the intervention (P0), the year during the intervention (P1), and the year after the intervention (P2). For the determination of drug costs, the costs for oral antidiabetic drugs and insulin were combined.

Physical activity was assessed via three items of the questionnaires, not using a validated physical activity scale:

1. On average, how many hours a day are you physically active? This includes activities that require moderate exertion and lead to a slight increase in breathing and pulse rate, such as going for a walk. Think about an average week.
2. On how many days in an ordinary week do you walk or ride a bicycle to get from one place to another, with a duration of at least ten minutes?
3. On an ordinary day, how much time do you invest in walking or bicycling from one place to another? (Indicate in minutes.)

For the intervention group, the number of steps per day was recorded using a pedometer at 12 months of intervention, but not afterwards.

Technology use was assessed in different ways at different points in time. At baseline, technology commitment was assessed using a validated scale; during the intervention, technology acceptance was assessed using a scale developed from a previous study and based on the technology acceptance model; and after the intervention, continued use of health-related technologies was assessed descriptively.

### Data analysis

The main analysis of primary and secondary outcomes was performed according to the intention-to-treat (ITT) principle using SPSS Version 26/28. For the ITT population, all randomised participants who answered the baseline questionnaire and submitted Hb_A1c_ values at T0 were included. Missing data were not replaced by imputations but handled indirectly using mixed models that provide valid statistical inference due to the “missing completely at random (MCAR)” assumption [49].

To represent baseline characteristics, mean values and standard deviations of the study participants were calculated for baseline. The presence of group differences at baseline was assessed via the Fisher exact test (dichotomous variables) or the Pearson X² test and the t-test (continuous variables).

Repeated measures linear mixed models were used for Hb_A1c_ Values and BMI separately using random intercept and fixed effects for the group (two levels), time (five levels), the interaction between both and the value at the outcome at baseline as covariate. Other covariates (e.g., age and gender) did not lead to model improvements and were therefore not included in the final model. A covariance structure based on autoregressive first order (AR1) could be selected for each model. The significance level was set to 0.05. Sensitivity analysis included non-responder imputation of missing data (baseline observation carried forward) and a per-protocol analysis of data. The per-protocol population was defined by finishing the complete 12 months of intervention regardless of missing intermediate values after 3 or 6 months, or lost to follow-ups after 24 months. We also determined how many individuals achieved the ADA-recommended criteria for both parameters during the course of the study.

For the analysis of physician contacts and medication costs, a small number of participants (N = 7) had to be excluded because, although they completed the study, they left the insurance company during the course of the study and thus their economic data could no longer be viewed. The available data did not show any missing values and were analysed using ANOVA with repeated measures and the group as a between-subjects factor.

The analysis of physical activity was divided into two parts. For both groups, we evaluated three questions from the questionnaire using unpaired T-tests for between-group differences. Furthermore, in a subgroup analysis, physical activity in the intervention group was additionally evaluated using an analysis of daily steps recorded during the 12-month intervention. Unfortunately, during the intervention, the transmission of step numbers from the participants decreased continuously, but at least 54% of participants (N = 47) from the IG submitted the number of daily steps for at least 9 out of the 12 months of intervention. Therefore, an evaluation with only these participants, and a second presentation with all existing cases and therefore with different N-numbers per month, were provided.

The surveys on technology use were evaluated descriptively.

## Results

### Baseline

**Fig. 1:**
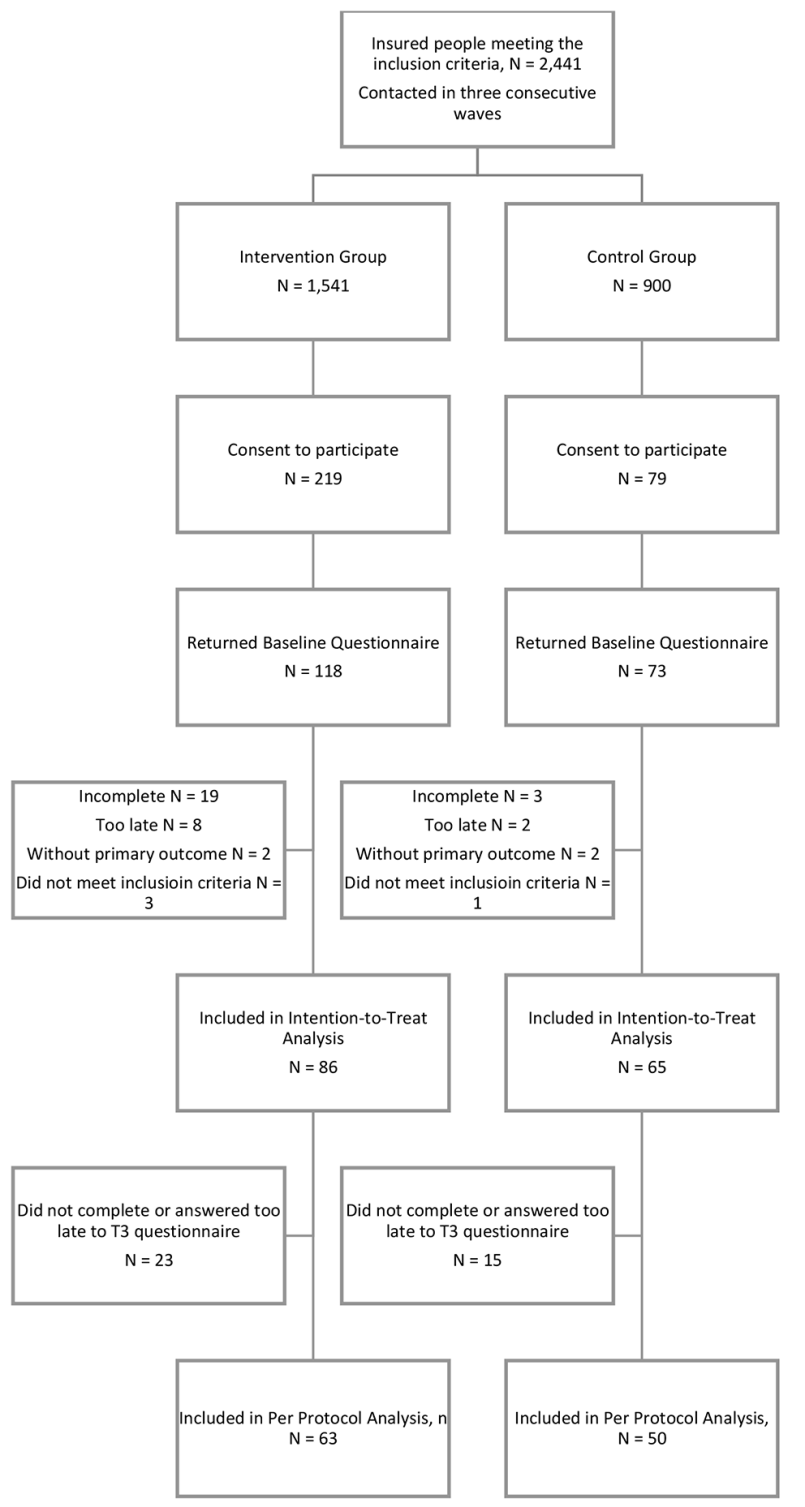
Consort Flow Chart

151 persons who answered the baseline questionnaire and submitted an Hb_A1c_ value at baseline were included in the study (Fig. 1), divided into the intervention group (IG; n = 86 people, 80.2% male, average age 59.7 years) and the control group (CG; n = 65 people, 83.1% male, average age 58.8 years). As presented in Table 1, most participants were men (n = 123, 81. 45%). The primary outcome Hb_A1c_ at baseline was 6.9% (SD 0.9) for the IG and 6.8% (SD 1.0) for the CG. Both groups showed no differences in demographic data, health history, or health parameters at baseline.

**Table 1:** Baseline characteristics

		*n*	Intervention Group	*n*	Control Group
Demographic Variables					
Age at study start (years)	M (SD)	86	59.66 (6.24)	65	58.80 (7.33)
Gender (male)	N (%)	86	69 (80.2)	65	54 (83.1)
Higher education (University degree)	N (%)	86	15 (17.4)	65	18 (27.7)
Higher income (> €5,000/month)	N (%)	59	11 (18.6)	53	15 (28.3)
Antidiabetic Medications					
Oral medication only	N (%)	86	54 (62.8)	65	43 (66.2)
Insulin only	N (%)	86	0 (0)	65	2 (3.1)
Both (oral and insulin)	N (%)	86	11 (12.8)	65	13 (20.0)
Health History					
Duration of diabetes (years since diagnosis)	M (SD)	84	6.29 (3.83)	65	6.91 (3.86)
Age at diagnosis	M (SD)	84	53.12 (7.09)	65	51.46 (7.22)
Multimorbidity (> 3 chronic diseases)	N (%)	86	84 (97.7)	65	64 (98.5)
Diabetes-related comorbidities	M (SD)	86	3.17 (1.96)	65	3.23 (1.99)
Health indicators					
Glycaemic control (Hb_A1c_)	M (SD)	86	6.94 (0.94)	65	6.83 (0.97)
BMI (kg/m²)	M (SD)	86	31.08 (7.05)	65	29.48 (4.53)
Physician contacts (in the year before the study)	M (SD)	72	10.78 (6.86)	61	11.61 (6.43)
Physical Activity					
> 3 h of moderate physical activity per day	N (%)	86	37 (43.1)	65	21 (32.3)

Data are mean ± standard deviation (SD) values or numbers (%) as indicated; ^*^P < .05 (using Fisher Exact Test for categorical variables and X² test and T-test for continuous variables, no significant differences found); BMI, body mass index; HbA1c, glycated haemoglobin

### Hb_A1c_ and BMI

**Fig. 2:**
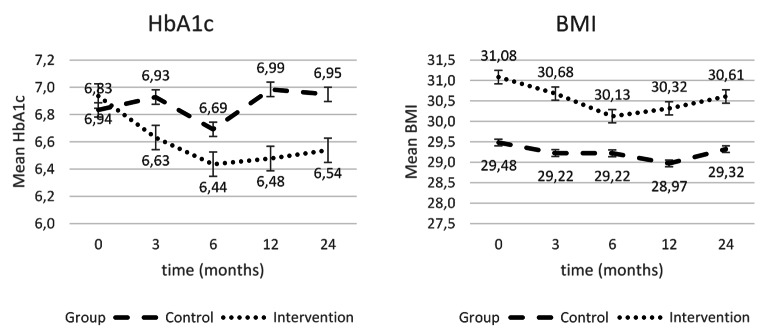
Development of Hb_A1c_ values and BMI over time; Data are the least-square means derived from a linear mixed model and adjusted for baseline haemoglobin A1c. Error bars indicate standard errors

At both ends of the intervention (-0.52 (-0.73; − 0.32), p < .000) and the 24-month follow-up (-0.38 (-0.61; − 0.15), p < .001), the intervention group showed significantly better Hb_A1c_ values compared to the control group (Table 2). When looking at the development over the entire period, it can be seen that the strongest decrease happened in the first three months, but the lowest value is reached only after six months (Fig. 2).

**Table 2:** Mean Changes in Glycemic Control (Hb_A1c_) and BMI in the Intervention and Control Groups across 12 months and 24 months

	Groups, Mean Change (95% CI)		Between Group Mean Difference (95% CI)	P Value of Difference
	Intervention Group (n = 86)	Control Group (n = 65)		
Hb_A1c_				
After 3 months	0.34 (0.19; 0.49)**	− 0.01 (-0.19; 0.16)	− 0.35 (-0.55; − 0.16)	0.000
After 6 months	0.51 (0.34; 0.68)**	0.27 (0.07; 0.47)*	− 0.24 (-0.45; − 0.03)	0.028
After 12 months	0.48 (0.31; 0.65)**	− 0.05 (-0.25; 0.15)	-,52 (-,73; -,32)	,000
After 24 months	0.39 (0.20; 0.58)**	0.01 (-0.21; 0.22)	-,38 (-,61; -,15)	,001
BMI				
After 3 months	0.49 (0.25; 0.72)**	0.05 (-0.23; 0.32)	− 0.43 (-0.81; − 0.05)	0.028
After 6 months	1.13 (0.85; 1.41)**	0.29 (-0.06; 0.63)	− 0.83 (-1.23; − 0.44)	0.000
After 12 months	1.33 (1.01; 1.64)**	0.25 (-0.12; 0.62)	-1,07 (-1,47; -,66)	,000
After 24 months	0.75 (0.39; 1.11)**	0.13 (-0.26; 0.53)	-,61 (-1,06; -,16)	,008

BMI, body mass index; Hb_A1c_, glycated haemoglobin,

Within-group change vs. Baseline * = <0.005 ** = < 0.000

Data are the least-square means derived from a linear mixed model and adjusted for baseline haemoglobin A1c. Error bars indicate standard errors

In terms of BMI, the intervention group showed significantly worse values in the group comparison after both 12 and 24 months, which, however, is due to the higher baseline value (Table 2). Looking at the time course, a significant reduction in BMI can be seen in the intervention group at both time points (12 M: 1.325 (1.011; 1.638), p < .000 24 M: 0.750 (0.389; 1.112), p < .000), which is not the case for the control group (Fig. 2).

There is also a significantly higher success rate as measured by target achievement of ADA recommendations for Hb_A1c_ levels in the intervention group, which is significantly different from the control group at both 12 and 24 months. However, this success does not hold true for BMI (Table 3).

**Table 3:** The proportion of Participants in Intervention and Control Groups who achieved the ADA Treatment Goal at Baseline, Year One and Year Two

	% of Group		
	Intervention Group	Control Group	P value between Group
Hb_A1c_			
Baseline	55,8	63,1	0.369
After 12 months	77,8	52,0	0.012
After 24 months	76,6	55,8	0.012
BMI			
Baseline	22,1	30,8	0.159
After 12 months	36,1	32	0.070
After 24 months	34	34,9	0.381

### Physician contacts and antidiabetic costs

A repeated-measures analysis of variance (assumed sphericity: Mauchly-W [2] = 0.992, p = .552) showed no significant main effect of time, but a significant interaction effect between time and group on the number of physician contacts (F(2,284) = 5.380, p = .005, ηp2 = 0.037). The effect size F according to Cohen (1988) is 0.196 and corresponds to a small effect. The analysis of the within-subjects contrasts showed that there was a significant change between the year before the intervention and the intervention year as well as the intervention year and the year after, but not between the year before the intervention and the year after the intervention, i.e., there was a regression to the baseline level. For costs, neither a significant main effect nor a significant interaction effect was found.

### Physical activity

**Fig. 3:**
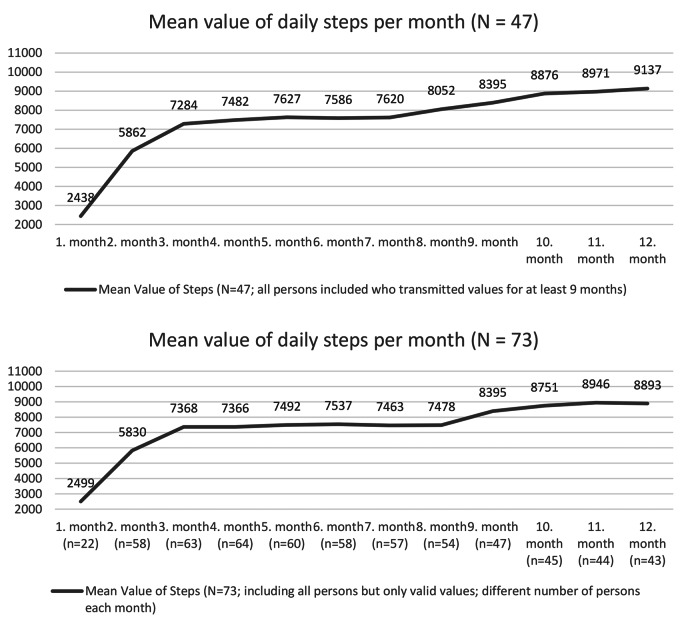
Values for Intervention Group Only – Daily Steps Recorded (Mean)

The analysis of the steps recorded shows a continuous increase over time in both plots (Fig. 3), so one can assume an increase in physical activity in the intervention group. In the analysis of the questions from the questionnaire, however, this is only reflected in one of the three questions, in which the intervention group also performs significantly better than the control group (Table 4).

**Table 4:** Results of Physical Activity Questions

		Intervention Group			Control Group			T-Test Between Group Effect					
		n	M	SD	n	M	SD	Mdiff	Sediff	T	df	p	d
Active hours per day	Baseline	86	3.5	1.0	65	3.2	1.1	− 0.28	0.17	-1.63	133,81	0.105	1.04
	12 M	62	3.8	1.1	50	3.4	1.0	− 0.38	0.20	-1.91	105.95	0.058	1.04
	24 M	47	3.6	1.0	43	3.3	0.8	− 0.32	0.19	-1.64	88	0.105	0.92
Active days (1–7)	Baseline	86	4.5	2.2	65	3.9	2.3	− 0.546	0.367	-1.458	149	0.140	2.24
	12 M	62	5.2	1.9	50	4.4	2.2	− 0.826	0.389	-2.088	97,1	.**039**	53.08
	24 M	47	4.6	2.3	43	4.2	2.3	− 0.390	0.483	− 0.808	88	0.421	2.05
min per Day	Baseline	86	50.9	59.2	65	44.4	43.7	-6.492	8.724	− 0.744	149	0.458	43.57
	12 M	62	52.3	45.0	50	51.6	41.7	− 0.706	8.281	− 0.085	110	0.932	2.29
	24 M	47	48.1	38.9	43	42.9	37.0	0.520	-5.178	− 0.645	88	0.521	38.04

### Use of technology

**Fig. 4:**
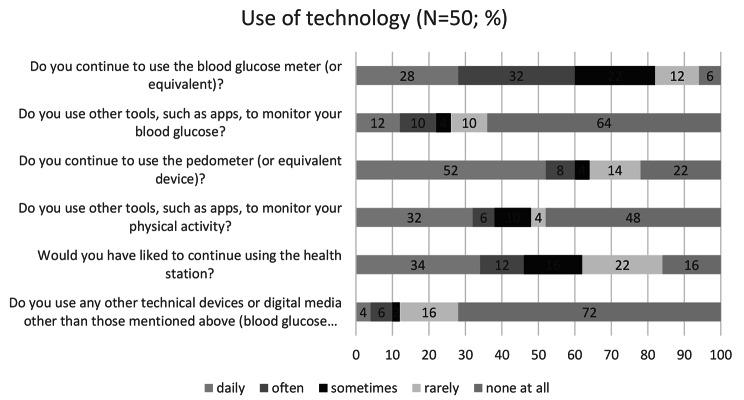
Use of technology after intervention

Both technology commitment and technology acceptance remain at a relatively high level throughout the study, so a high acceptance rate and few problems in use can be assumed (Table 5). The questionnaire on continued use after the end of the intervention shows that 60% of the respondents continue to use the devices or equivalents regularly (Fig. 4).

**Table 5:** Technology acceptance of Intervention group

	T1 (N = 60)		T2 (N = 62)		T3 (N = 62)	
	M	SD	M	SD	M	SD
Perceived ease of use	4.48	0.69	4.62	0.59	4.65	0.59
Perceived usefulness	4.27	0.67	4.41	0.81	4.48	0.76
Technology self-efficacy	4.73	0.54	4.74	0.52	4.81	0.36
Relevance to everyday life	4.57	0.68	4.53	0.87	4.59	0.78
Perceived enjoyment	3.89	1.00	3.75	1.19	4.00	1.08
Subjective norm	4.38	0.82	4.49	0.65	4.40	0.89
Feeling of being controlled	4.14	1.04	3.99	1.21	4.30	1.05
Sense of security	4.14	0.89	4.30	0.96	4.31	1.04

Data are mean ± standard deviation (SD) values according to the Per Protocol sample of the intervention group. Values represent scores from 1 (no agreement) to 5 (full agreement)

## Discussion

This study has shown significant improvements in Hb_A1c_ levels over two years for participants in the initiative.diabetes programme in addition to standard care. The use of telemedicine coaching in combination with various wearables, therefore, offers a good option for the long-term treatment of patients with type 2 diabetes. The telemedicine-supported coaching on the management of T2DM, proper diet, and appropriate exercise manifested itself very clearly in the clinical outcomes of Hb_A1c_ and BMI. Values of the primary outcome Hb_A1c_ were significantly lower in the intervention group (IG) than in the control group (CG) at all measurement time points. This strongest change was seen in the first three months, which is consistent with the positive short-term results of many studies. However, it is also evident that the lowest value was reached only after six months, so three months of intervention are not sufficient to achieve optimal results. The second six months of the intervention showed little change, which can be attributed to the programme structure based on Prochaska’s transtheoretical model [42–44]. In the first six months, behaviours were to be changed, while in the stabilisation phase, the aim was to integrate them into everyday life in such a way that they could be implemented in the long term beyond the end of the programme. This is shown in the follow-up survey, in which the intervention group also performs significantly better.

In addition, it should be noted that, according to the recommendations of ADA [50] and EASD [20], an Hb_A1c_ value of less than 7% for type 2 diabetics should be targeted in the long term for nonpregnant adults. In the present study, 55% of the intervention group and 63% of the control group had already achieved Hb_A1c_ values below the recommended 7% at the beginning of the study, which also leads to baseline values just below 7% on average in both groups. Despite the low baseline value, a significant reduction was achieved, demonstrating a clear positive effect of the intervention. With higher baseline values, an even more significant result would have been expected. This is consistent with the results of one of the few large long-term studies of intensive lifestyle interventions (ILI), the Look AHEAD study [21, 51]. Here, the Hb_A1c_ value in the ILI group was reduced by 0.7%, from 7.3 to 6.6%, within one year. The corresponding values from the present study are a reduction of about 0.5% from a baseline of about 6.9%, so that in the intervention group nearly 78% are now achieving the ADA recommendation, while in the control group, the proportion has become smaller. Lifestyle changes can thus bring about extremely positive changes even in a rather mild stage of T2DM. These changes have a positive effect not only on diabetes control. For example, it is also assumed that the control of DM contributes to the significant reduction of other non-communicable diseases, such as dementia [52]. According to the UK Prospective Diabetes Study, a 1% absolute reduction in Hb_A1c_ (equivalent to 11 mmol/mol) over 10 years can translate into a 21% reduction in diabetes-related endpoints and a reduction in microvascular complications of approximately 37% [53]. However, such high reductions are rarely achieved. In the literature, values between 0.25% and 0.5% (corresponding to 3–5 mmol/mol) are most common immediately after completion of the intervention and may already make a significant difference with regard to the further development of diabetes [38, 54, 55]. In the present study, this reduction was achieved both at the end of the intervention and in the follow-up survey.

At the follow-up survey one year after the end of the intervention, the intervention group has an average Hb_A1c_ value of 6.54%, which is still below the international recommendations of the ADA and EASD, and significantly below the value of the control group. The decrease over the entire year following the intervention, during which there was no further support outside of regular care, was only 0.1%. This sustained improvement demonstrates the long-term effectiveness of the intervention.

The participants’ BMI also showed a significant reduction of -1.370 (CI -1.822; − 0.919, p < .000) after 12 months and a permanent downward trend in the IG. Compared to the international Look AHEAD study [51] mentioned above or the German TeliPro study [23], this is a smaller reduction, but in both studies, diet products were explicitly used for weight reduction. In this study, participants were offered support via dietary products. However, these were not a mandatory component and were only used by one person in the included study sample. Therefore, the results are predominantly due to the coaching and resulting lifestyle adjustments.

Weight management measured by BMI did not show such good results as Hb_A1c_. Although a positive development of the intervention group was recognisable after the intervention, this could not be maintained by the patients. One year after the end of the intervention, the participants in the intervention group are, on average, relatively close to baseline again, or at least show a significant regression in intervention success. This is not surprising, as it has been shown in many studies that lost weight is regained after the intervention ends, and patients often go through several cycles of weight loss and relapse until they achieve constant weight management [56–59]. In general, studies in the area of weight management often report rather disappointing results and lower effectiveness, especially due to poorer attendance and adherence rates.

It has also been shown that extending the support beyond the intervention period leads to better consolidation of the effects, and longer intervention durations achieve better results in weight loss than shorter ones [60, 61]. International guidelines of the ADA recommend, in the area of weight management for type 2 diabetics, at least one year of further support with at least one contact per month and appropriate self-monitoring after a six-month intensive coaching phase, so that long-term successes can be maintained [62]. In the present study, after six months of intensive coaching there were only six months for the stabilisation phase, which could be partly responsible for the stronger regression in this area. In contrast to this, the descriptive survey of the patients (Fig. 4) showed that only a small proportion of the participants would have liked to receive longer care. Here, the patients’ interests do not coincide with the procedures recommended in the literature. In addition, a lower baseline is considered a predictor of better weight loss success and adherence [63]. In the current study, baseline values were already above the general recommendations on average, which is not surprising given that overweight or obesity are some of the most common side effects in type 2 diabetics.

Finally, a study of various contact options found that support via telephone or the internet is not as successful in the area of weight management as personal support [64]. This is not the case in other areas. In the area of blood glucose control, for example, some studies show that interventions carried out via telemedicine are no less effective than interventions carried out conventionally [38].

The number of physician contacts showed a significant increase in the intervention group in the year of the intervention but fell back to the baseline value in the following year. This could be due to increased healthy behaviour, increased controls or changes in medication. However, since only the number and not the reasons for the visits to the doctor were recorded, a bias cannot be ruled out here. The evaluation of medication costs showed no significant changes in either group, which is most likely due to the study duration being too short for actual economic evaluations.

The descriptive analysis of the transmitted daily steps also shows an increase in physical activity within the intervention group. No step counts were submitted by the control group, so a comparison is not possible here. On average, the participants included in the analysis achieved between 8,000 and 9,000 steps, and are thus relatively close to the recommended 10,000 steps per day [65]. In addition to numerous other determinants, a physically active lifestyle is considered to have a major impact on good physical and mental health, a defence against chronic diseases, the maintenance of independence and mobility, and improved everyday skills up to an advanced age [66–68]. In the preventive area of public health research, numerous studies have confirmed that physical activity reduces next to the risk of T2DM the risk of early death, coronary heart disease, stroke, hypertension, metabolic syndrome, colon and breast cancer, and depression [68]. However, especially at the end of the intervention, many participants no longer transmitted values, so the evaluation of the recorded steps must be viewed with caution and a certain bias cannot be ruled out. Reasons for the lack of data transmission can be interpreted positively or negatively. Negative reasons could be technical problems or lack of motivation. However, since both technical commitment and technology acceptance were very high throughout the entire intervention period, it seems rather unlikely that technical problems were the main reason. Nevertheless, the motivational component of wearing the pedometer constantly every day may have played a role. Another, more positive explanation could be that the structural changes were already integrated and consolidated so well into everyday life that the visualisation could no longer offer any significant benefit to the participants and was no longer needed towards the end of the intervention. This would also be reflected in the fact that no more significant changes in outcomes occurred towards the end of the intervention; instead, the values remained largely stable. An increase in physical activity in the intervention group can nevertheless be assumed, since this was also shown in the questions recorded simultaneously in the questionnaire. Unfortunately, the increase in physical activity is reflected only slightly and not statistically significantly in the evaluation of the questionnaire (with the exception of the group comparison of active days per week). The recording of physical activity by means of questionnaires is generally considered difficult and will require increased research in the future. In particular, lighter physical activities, which make up the largest part of daily physical activity, are difficult to record via subjectively answered questionnaires [69].

A major advantage of the programme’s telemedicine approach was also evident during the current COVID-19 pandemic, which had its first peak phase in the last six months of the study and is still ongoing. The programme was able to continue without restructuring measures and all patients could be cared for as usual due to the lack of physical dependence on care centres, hospitals or similar. The high flexibility of the programme has also proven to be very useful when it came to balancing family and work with the coaching. This continuity of the programme is an important positive factor, especially in the ongoing process of behavioural change. The programme’s flexibility, through individual and needs-oriented coaching, also showed clear positive effects. Many patients showed reduced physical activity due to numerous restrictions on leisure activities during the pandemic. In this case, the coaches were able to react individually and motivate the participants to exercise more in their protected home setting. For this purpose, exercise mailings with instructions or similar offers were sent out to the participants. This shows the enormous importance of so-called two-way communication, where one can interact with a human counterpart. In addition, the programme offers patients the possibility of contacting the coach at any time if there is a need, for example, due to changed structures or crisis-related developments in their own behaviour.

Overall, telemedicine-supported coaching with two-way communication proved to be very effective. With further coaching, these results were maintained at a stable level in the second half of the intervention. In practice, these results show that programmes aimed at changing the patient’s behaviour or habit structures should have a duration of at least 6 months, preferably up to 12 months, to stabilise the achieved effects. Whether these good results remain stable after another year without coaching will be shown by a later evaluation of the 24-month follow-up.

### Strengths and limitations

The major limitation of the present study is the lack of representativeness due to the sampling being restricted to the population of insured persons of a private health insurance company.

In the German healthcare system, a distinction is made between public and private health insurance, with people with a higher level of education and higher income usually choosing private health insurance [70–72]. Furthermore, their higher socioeconomic status could have an impact on the success of the programme, since on the one hand, a better understanding of the necessity of changes in one’s own lifestyle can be assumed, and on the other hand, on average, there are sufficient financial resources available to fund such changes, e.g., the cost of sports club memberships, healthy and therefore often more expensive food, etc. It may also play a role in their already very high acceptance of technology and readiness to use technology. The gender distribution, however, is not likely to matter too much, as the general distribution of diabetes prevalence also shows slightly more males than females, although with a slightly smaller difference than in the present study [4].

A second limitation could be the randomisation strategy, since the participants were first randomised and then invited to participate in a specific group. Therefore, a certain motivation bias could be present. Nevertheless, no significant differences were found in the baseline.

Strengths of the study, on the other hand, are the long duration as well as the follow-up and the fact that it is a randomised controlled trial.

## Conclusion

Overall, the telemedicine-supported coaching programme initiative.diabetes with two-way communication proved to be very effective. With further coaching, these results were maintained at a stable level in the second half of the intervention and were also established in the long term. For practical purposes, these results show that programmes aimed at changing a patient’s behavioural or habitual structures should have a duration of at least 6 months, preferably up to 12 months, to stabilise the achieved effects.

Furthermore, for future studies, the focus should be on concepts for long-term establishment in the patients’ everyday lives and the evaluation of long-term effects. In addition, a future study should investigate the roles of the coaching and the telemedical wearables separately.

## List of abbreviations

ADA: American Diabetes Association
AR1: Auto-regressive first order
BMI: Body mass index
CG: Control group
DM: Diabetes Mellitus
EASD: European Association for the Study of Diabetes
IDF: International Diabetes Federation
IG: Intervention group
ILI: Intensive lifestyle interventions
ITT: Intention-to-Treat
MCAR: Missing completely at random
P: Period
SD: Standard deviation
T2DM: Type 2 Diabetes
